# Editorial Comment: Ultra-hypofractionated versus conventionally fractionated radiotherapy for prostate cancer: 5-year outcomes of the HYPO-RT-PC randomised, non-inferiority, phase 3 trial

**DOI:** 10.1590/S1677-5538.IBJU.2020.06.09

**Published:** 2020-09-02

**Authors:** Felipe Lott

**Affiliations:** 1 Instituto Nacional do Câncer Rio de JaneiroRJ Brasil Instituto Nacional do Câncer – INCA, Rio de Janeiro, RJ, Brasil

Anders Widmark ^1^, Adalsteinn Gunnlaugsson ^2^, Lars Beckman ^3^, Camilla Thellenberg-Karlsson ^4^, Morten Hoyer ^5^, Magnus Lagerlund ^6^, et al.

^1^ Department of Radiation Sciences, Oncology, Umeå University, Umeå, Sweden; ^2^ Department of Hematology, Oncology and Radiation Physics, Skåne University Hospital, Lund University, Lund, Sweden; ^3^ Department of Oncology, Sundsvall Hospital, Sundsvall, Sweden; ^4^ Department of Radiation Sciences, Oncology, Umeå University, Umeå, Sweden; 5 Department of Oncology and Danish Centre for Particle Therapy, Aarhus University Hospital, Aarhus, Denmark; 6 Department of Oncology, Kalmar Hospital, Kalmar, Sweden

Lancet. 2019 Aug 3;394(10196):385-395.

**DOI: 10.1016/S0140-6736(19)31131-6 | ACCESS: 10.1016/S0140-6736(19)31131-6**

## COMMENT

Due the low alpha/beta ratio, the hypofractionation of the external radiotherapy treatment of prostate cancer can increase the therapeutic ratio and reduce the health-care cost and improve the patient comfort. It can be done by moderate hypofractionation (using 2.4 – 3.4 Gy) or by ultra-hypofractionation (at least 5 Gy per fraction) ( 1 - 3 ).

This phase 3 non-inferiority randomized trial is the first to report on the efficacy and side-effects on ultra-fractionation compared with conventional and has the PSA relapse and clinical failure as primary endpoint. The most relevant secondary endpoints were the overall survival and prostate cancer-specific survival and the median follow-up time was 5yr.

The ultra-hypofractionation was non-inferior to the conventional fractionation (HR 1.002) and no significant differences were found in terms of relevant urinary or gastrointestinal toxicity.
